# Infant feeding practices and breastfeeding duration in Japan: A review

**DOI:** 10.1186/1746-4358-7-15

**Published:** 2012-10-25

**Authors:** Madoka Inoue, Colin W Binns, Keiko Otsuka, Masamine Jimba, Manami Matsubara

**Affiliations:** 1 School of Public Health, Curtin Health Innovation Research Institute, Curtin University, GPO Box U1987, Perth, Western Australia 6845, Australia; 2 Department of Community and Global Health, Graduate School of Medicine, University of Tokyo, 7-3-1, Hongo, Bunkyo-ku, Tokyo, 113-0033, Japan; 3 School of Nursing, St. Mary College, 422, Tsubukuhonmachi, Kurume City, Fukuoka, Japan

## Abstract

The Japanese health system places great emphasis on healthy development. However, the prevalence of Exclusive Breastfeeding at one month postpartum between 1980 and 2005 has remained unchanged, fluctuating between 42% and 49%. At the same time, the Any Breastfeeding prevalence has gradually increased from about 80% to 95%. In 2010, the latest national breastfeeding report showed that ‘exclusive’ and ‘any’ breastfeeding rates have improved. However, as the World Health Organization (WHO) definition of breastfeeding practices was not used in this study or in other national surveys, it is difficult to interpret these latest results. While the Japanese government has launched several promotion projects, there have been few studies and reviews of risk factors that influence breastfeeding duration. The objectives of this review were to summarise the factors that have influenced the duration of breastfeeding in Japan to provide information relevant to breastfeeding promotion programs. A search of electronic databases in Japanese and English was undertaken up to 2011. The inclusion criteria for this review were studies that focused on infant feeding practices and targeted Japanese mothers, fathers, or health professionals, but excluded mothers’ friends and peer groups. In total, 12 articles were selected for the final analysis. Smoking status, low birth weight of infants and maternal perceptions of insufficient breast milk supply were negative influences on breastfeeding duration, while support from husbands/partners is associated with continued breastfeeding. Some factors that have been found to be associated with breastfeeding in other countries, including maternal age, family income, maternal educational levels, and living with grandparents of infants have not been confirmed in Japan. While the national breastfeeding rates were higher than other countries of similar health status, inconsistent knowledge of breastfeeding benefits and inappropriate hospital practices remain in Japan may be associated with increased the use of infant formula and reduced breastfeeding duration. Most of the studies reviewed were cross-sectional in design, with only a limited number of cohort studies. Also many published studies used small sample sizes. Cohort studies of infant feeding practices with larger sample sizes are required to monitor trends in rates and risk factors for breastfeeding outcomes.

## Background

The importance of breastfeeding has been documented in numerous scientific studies, including recent major reviews [1-3]. Breastfeeding, particularly Exclusive Breastfeeding for the initial six months of life, provides better health for both infants and mothers by preventing diseases and promoting health in the short and long term [4]. There has been an emphasis on the promotion of breastfeeding in many countries and in 2001 the Japanese government undertook a health promotion project, ‘Healthy and Happy Family 21 (Sukoyaka Oyako 21)’ that included a goal to improve the rate of Exclusive Breastfeeding at one month postpartum by 2014. The 2010 interim report on this project stated that although more than 95% of women intended, before delivery, to breastfeed their infants, the rate of Exclusive Breastfeeding at one month postpartum had not changed since 2005 [5,6]. No specific target was set at the beginning of this project, making it hard for health professionals to evaluate if the goal was achieved. Subsequently in 2010, a target was set of 60% Exclusive Breastfeeding at one month postpartum [5].

**Figure 1:**
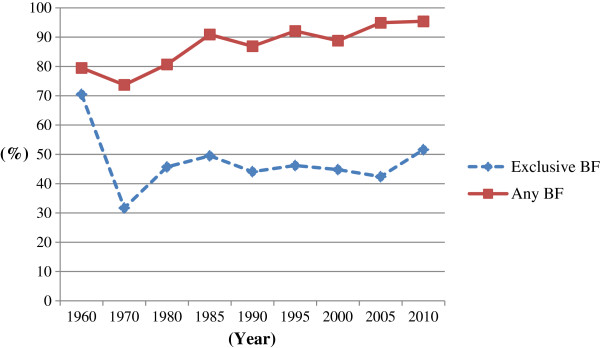
**The breastfeeding rate at one month after birth in Japan from 1960 to 2010. **Footnote: Source: from 1960 to 2005: [12], 2010:[13]. *BF = breastfeeding. ‘Exclusive Breastfeeding’ should be considered as ‘full or Predominant Breastfeeding’ due to the differing definitions of breastfeeding status used in Japan.

In Japan, Exclusive Breastfeeding was the only practical feeding method to feed infants until the end of the first half of the 20th century [7]. For women, visiting temples or shrines to pray for their ability to have sufficient production of breastmilk was common practice and many special foods were consumed to help with lactation [8]. A child usually continued breastfeeding until two to three years of age, and breastfeeding up to six years of age was common [7]. Infant formula was first introduced to Japan in 1917, but it was not until the 1950s that its use increased rapidly [9]. Formula companies used the advertising slogan ‘To become a bright child’ in their marketing, and this message suggested that infant formula feeding gave advantages in infant development and weight gain, which increased its popularity among mothers [10]. The Exclusive Breastfeeding rate declined to its lowest point of 31% at one month in 1970 [Figure 1. As a result, in 1975, three goals for restoring breastfeeding rates were set by the Ministry of Welfare based on the 1974 recommendations of the WHO. These were:

1) To exclusively breastfeed infants to 1.5 months after birth

2) To exclusively breastfeed infants to three months if possible, and

3) Not to change to formula, even after four months of age, without good reasons [11]

**Figure 2:**
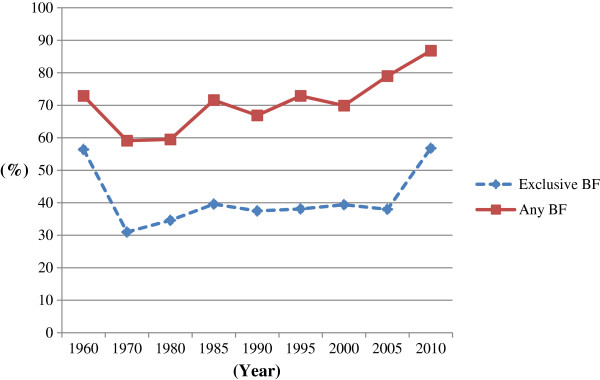
**The breastfeeding rate at three months after birth in Japan from 1960 to 2010. **Footnote: Source: from 1960 to 2005: [12], 2010:[13]. *BF = breastfeeding. ‘Exclusive Breastfeeding’ should be considered as ‘full or Predominant Breastfeeding’ due to the differing definitions of breastfeeding status used in Japan.

The Any Breastfeeding rate has gradually increased since then. The National Nutritional Survey of Pre-school Children, which has been carried out every decade since 1985, showed that Any Breastfeeding has increased from 80.7% in 1980 to 94.9% in 2005 at one month and from 59.5% to 79.0% at three months, while the duration of Exclusive Breastfeeding has remained relatively constant, but with a slight decline in recent years [Figures 1 and 2[6]. The rate of Exclusive Breastfeeding at one month declined from 49.5% in 1985 to 46.2% in 1995 and to 42.2% in 2005 [Figure 1[6]. The rate at three months has also remained relatively constant at 39.5% in 1985, 38.1% in 1995 and 38.0% in 2005 [Figure 2[6]. In 2010, another National Survey on Children’s Growth reported that the rate of Exclusive Breastfeeding was 51.6% at one month [Figure 1 and 56.8% at three months [Figure 2[13]. In this survey, Any Breastfeeding also increased to 95.4% at one month and 86.8% at three months. Compared with previous reports of breastfeeding rates in Japan, the latest rates of Exclusive Breastfeeding have improved. However, the WHO definitions of breastfeeding practices were not applied in this study and therefore it is difficult to compare 2010 results with international studies. While the Japanese government set three goals for mothers in the first breastfeeding promotion campaign in 1975 (see above), the rate of Exclusive Breastfeeding has remained unchanged for the last 30 years.

Japan was the first developed country to have a Baby-Friendly Hospital Initiative (BFHI) hospital accredited by the WHO and United Nations Children’s Fund (UNICEF) in 1991 [14]. However, Japan was also one of the three countries that abstained from voting for ‘International Code of Marketing of Breastmilk Substitutes’ in 1981 [9]. These contradictive actions suggested to Japanese health professionals that perhaps breastfeeding promotion was not a high priority and not an issue that should be applied to the whole population. Studies from other countries have found that factors that influence breastfeeding outcomes include maternal age, maternal infant feeding attitudes, breastfeeding difficulties, maternal employment status, and maternal smoking status [15-17]. However, there have been few studies published on the factors associated with breastfeeding duration in Japan. The aim of this review is to summarise factors that influence the duration of breastfeeding in Japan and the existing knowledge of breastfeeding that will assist the development of further promotion programs.

The standard WHO definitions of breastfeeding used in this review are [18]:

• Exclusive Breastfeeding: Infants receive breastmilk. It precludes the use of any other liquids or solids since delivery, other than specific medications.

• Predominant (full) Breastfeeding: Infants receive breastmilk as the main nourishment and are also allowed to have water, water-based drinks, fruits juice, and ORS. However, it precludes the use of formula and solids.

• Any (complementary) Breastfeeding: Infants receive some breastmilk and may also receive infant formula with or without solids.

## Methods

**Figure 3:**
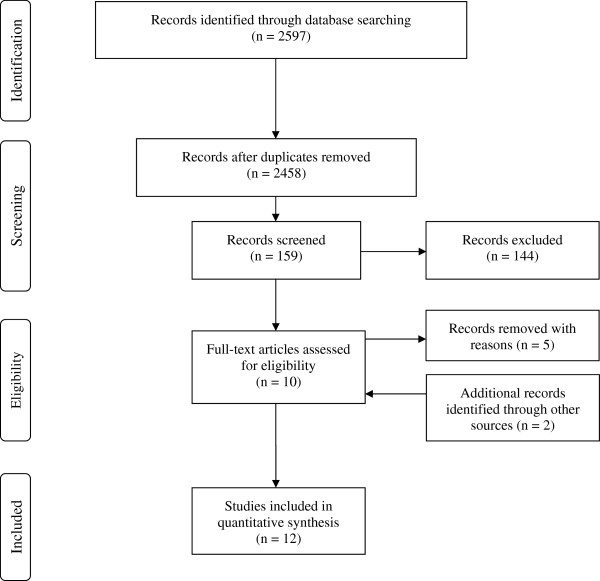
Selection process of the studies reviewed in this paper.

A literature search was undertaken using the electronic databases, PubMed, Proquest, Web of Science, ICHUSHI, J-STAGE, and CiNii using the key-words of ‘breastfeeding’, ‘breast-feeding’, ‘factor(s)’, ‘determinant(s)’, ‘breastfeeding duration’ ‘fathers (spouse/partners)’ ‘Japan’, ‘Japanese’, and ‘infant feeding’. These key-words were used in combination with the Boolean operators ‘AND’ or ‘OR’ to search literature and duplicate records were then removed. Irrelevant titles and non-Japanese samples were also removed. After abstracts were reviewed, relevant full-text papers were obtained but irrelevant contents including qualitative studies were again removed. The references in the relevant full-text papers were checked as other sources and included if necessary. The PRISMA 2009 flow diagram [19] describes the process used for searching the relevant literature [Figure 3. The inclusion criteria for this review were infant feeding practices in Japan and the studies that focused on Japanese mothers, fathers or health professionals. All English and Japanese papers were selected for this review and there were no limitations on the year of publication, to the end of 2011. The exclusion criteria were any papers targeting Japanese mothers’ friends.

## Results

In total, 12 articles were reviewed. The factors are categorized into maternal, infant, and socio-environmental attributes. The available data is summarized in Table 1.

**Table 1:** Japanese studies dealing with factors that influence the duration of breastfeeding

**Factors**	**Authors (Year)**	**Study design**	**Sample size (n)**	**Definition of breastfeeding**	**Exclusive Breastfeeding rate (%)**	**Crude OR (95% CI) (subgroup)**	**Adjusted OR (95% CI) (subgroup)**	**Study details**
**Maternal age**	Kaneko et al. (2006) [20]	Cross-sectional	45,569	1. Breastfed	22.4 at 6m	1.2 (1.14,1.26) (30-39 years old)	0.89 (0.84,0.94) (30-39 years old)	Mothers who are between 20-29 years old = 1 (reference)
				2. Only colostrum				
				3. Not breastfeed				
	Yokoyama et al. (2006) [21]	Cross-sectional	15262	1. Breastfeeding only	N/A	N/A	N/A	
				2. Mixed-feeding (mainly breast with some bottle or mainly bottle with some breast)				
				3. Bottle feeding with formula milk only				
	Otsuka et al. (2008) [22]	Cross-sectional	262	1. Exclusive Breastfeeding (i.e. breastmilk only)	40 at 4wks	5.37 (1.18,24.40)	N/A	
				2. Partial breastfeeding (i.e. breastmilk and formula)				
				3. Bottle-feeding (i.e. no breastmilk at all)				
	Sasaki et al (2009) [23]	Longitudinal	908	1. Only breastfeeding	27.6 at 3-4m	N/A	N/A	Not statistically significant (p = 0.93)
				2. Breastmilk and infant formula				
				3. Only infant formula				
**Marital status**	Otsuka et al. (2008) [22]	Cross-sectional	262	1. Exclusive Breastfeeding (i.e. breastmilk only)	40 at 4wks	N/A	N/A	Not statistically significant (p = 0.88)
				2. Partial breastfeeding (i.e. breastmilk and formula)				
				3. Bottle-feeding (i.e. no breastmilk at all)				
**Socioeconomic status**	Kaneko et al. (2006) [20]	Cross-sectional	45,569	1. Breastfed	19 at 6m (< 3.9 million yen) 22 at 6m (> 8.0 million yen)	0.85 (0.80,0.90) (< 3.9 million yen) 1.03 (0.97,1.08) (> 8.0 million yen)	0.93 (0.88,0.99) (< 3.9 million yen) 1.03 (0.97,1.09) (> 8.0 million yen)	4.0-7.9 million yen of annual income = 1 (reference)
				2. Only colostrum				
				3. Not breastfeed				
	Otsuka et al. (2008) [22]	Cross-sectional	262	1. Exclusive Breastfeeding (i.e. breastmilk only)	40 at 4wks	1.0 (0.26,3.55) (>2,000,000 yen)	N/A	Not statistically significant (p = 0.96)
				2. Partial breastfeedin (i.e. breastmilk and formula)				
				3. Bottle-feeding (i.e. no breastmilk at all)				
**Educational status**	Otsuka et al. (2008) [22]	Cross-sectional	262	1. Exclusive Breastfeeding (i.e. breastmilk only)	40 at 4wks	1.3 (0.66,2.49) (High school or less)	N/A	Not statistically significant (p = 0.47)
				2. Partial breastfeeding (i.e. breastmilk and formula)				
				3. Bottle-feeding (i.e. no breastmilk at all)				
**Maternal employment status**	Kaneko et al. (2006) [20]	Cross-sectional	45,569	1. Breastfed	25.0 at 6m	1.17 (1.09,1.26) (Full time and child care leave for 6m or more)	1.14 (1.05,1.23) (Full time and child care leave for 6m or more)	Non-workers = 1 (reference)
				2. Only colostrum				
				3. Not breastfeed				
	Sasaki et al. (2009) [23]	Longitudinal	908	1. Only breastfeeding	27.6 at 3-4m	N/A	N/A	Not statistically significant (p = 0.12)
				2. Breastmilk and infant formula				
				3. Only infant formula				
**Multiple births**	Kaneko et al*.* (2006) [20]	Cross-sectional	46,569	1. Breastfed	1.3 at 6m	0.05 (0.03,0.09)	0.07(0.04,0.12)	Single birth = 1 (reference)
				2. Only colostrum				
				3. Not breastfeed				
	Yokoyama et al. (2006) [21]	Cross-sectional	15,262	1. Breastfeeding only	N/A	N/A	N/A	Multiple births was associated with breastfeeding cessation (adjusted OR = 0.07, 95% CI = 0.04,0.12)
				2. Mixed-feeding (mainly breast with some bottle or mainly bottle with some breast)				
				3. Bottle feeding with formula milk only				
	Ooki (2008) [24]	Cross-sectional	4,023	1 Full breastfeeding	6.6 at 6m (Full breastfeeding)	N/A	N/A	Sample were mother who had twin. Any Breastfeeding and formula feeding at 6m were 36.1 and 54.1%, respectively
				2. Partial breastfeeding (mixed feeding)				
				3. Formula feeding				
**Maternal smoking status**	Kaneko et al. (2006) [20]	Cross-sectional	46,324	1. Breastfed	11.5 at 6m	0.40 (0.31,0.95)	0.44 (0.34,0.57)	Nil smoking of the mothers and fathers = 1 (reference)
				2. Only colostrum				
				3. Not breastfeed				
	Sasaki et al. (2009) [23]	Longitudinal	908	1. Only breastfeeding	27.6 at 3-4m	N/A	N/A	Not statistically significant (p = 0.25)
				2. Breastmilk and infant formula				
				3. Only infant formula				
**Delivery methods**	Nakao et al. (2008) [25]	Cross-sectional	318	1. Fully breastfeeding (breastmilk was given and infant formula was not given, regardless of whether other liquids and/or solid food were given)	N/A	N/A	1.02 (0.41,2.59)	Not statistically significant (p = 0.96)
				2. Any Breastfeeding (breastmilk and infant formula were given regardless of whether other liquids and/or solid food were given)				
				3. Infant formula feeding (formula was given and breast milk was not given regardless of whether other liquids and/or solid food were given)				
	Sasaki et al. (2009) [23]	Longitudinal	908	1. Only breastfeeding	N/A	N/A	N/A	Not statistically significant (p = 0.14 for chi square test)
				2. Breastmilk and infant formula				
				3. Only infant formula				
**Parity**	Kaneko et al*.* (2006) [20]	Cross-sectional	45,569	1. Breastfed	24.5 at 6m (Second times) 26.1at 6m (Third times or more)	1.57 (1.49,1.65) (Second times) 1.71 (1.60-1.81) (Third times or more)	1.72 (1.63,1.81) (Second times) 2.06 (1.91-2.22) (Third times or more)	Primipara = 1 (reference)
				3. Not breastfeed				
				2. Only colostrum				
	Otsuka et al. (2008) [22]	Cross-sectional	262	1. Exclusive Breastfeeding (i.e. breastmilk only)	40 at 4wks	N/A	N/A	Not statistically significant (p = 0.90)
				2. Partial breastfeeding (i.e. breastmilk and formula)				
				3. Bottle-feeding (i.e.no breastmilk at all)				
	Sasaki et al. (2009) [23]	Longitudinal	908	1. Only breastfeeding	N/A	N/A	N/A	Not statistically significant (p = 0.55)
				2. Breastmilk and infant formula				
				3. Only infant formula				
**Current intake of alcohols**	Sasaki et al. (2009) [23]	Longitudinal	908	1. Only breastfeeding	N/A	N/A	1.37 (1.02,1.86)	Not intake of alcohols = 1 (reference) (p = 0.04)
				2. Breastmilk and infant formula				
				3. Only infant formula				
**Early initiation of breastfeeding**	Nakao et al. (2008) [25]	Cross-sectional	318	1. Fully breastfeeding (breastmilk was given and infant formula was not given, regardless of whether other liquids and/or solid food were given)	N/A	N/A	2.45 (1.21,4.95)	Initiating breastfeeding within 120 minutes after birth was positively associated with duration of breastfeeding at 4 months (p = 0.01)
				2. Any Breastfeeding (breastmilk and infant formula were given regardless of whether other liquids and/or solid food were given)				
				3. Infant formula feeding (formula was given and breast milk was not given regardless of whether other liquids and/or solid food were given)				
**Intention to breastfeed**	Nakamura et al. (2002) [26]	Cross-sectional	105	1. Breastfeeding	73.9 at 3m	N/A	N/A	
				2. Breast & Bottle feeding				
				3. Artificial milk				
	Otsuka et al. (2008) [22]	Cross-sectional	262	1. Exclusive Breastfeeding (i.e. breastmilk only)	40 at 4wks	N/A	N/A	Intention to Exclusive Breastfeeding = 1 (reference)
				2. Partial breastfeeding (i.e. breastmilk and formula)				
				3. Bottle-feeding (i.e. no breastmilk at all)				
**Maternal attachment**	Sasano et al. (2005) [27]	Cross-sectional	182	1. Breastfeeding	53.4 at 3m	N/A	N/A	
				2. Mix and infant formula feeding				
**Breastmilk insufficiency**	Yoshitome et al. (2003) [28]	Cross-sectional	246	1 Breastfeeding only	38.5 at 3-4m	N/A	N/A	
				2. Mix feeding				
				3. Infant formula only				
**Low birth weight**	Kaneko et al*.* (2006) [20]	Cross-sectional	46,557	1. Breastfed	11.4 at 6m	0.46 (0.42,0.51)	0.67 (0.60,0.76)	≥ 2500g =1 (reference)
				2. Only colostrum				
				3. Not breastfeed				
**Maternal confidence**	Awano et al. (2010) [29]	quasi-experimental	117	1 Fully breastfeeding (no formula was given)	72.2 at 4 wks (Full breastfeeding)	N/A	N/A	Breastfeeding self-care program is effect to improve maternal confidence
	Otsuka et al. (2008) [22]	Cross-sectional	262	1. Exclusive Breastfeeding (i.e. breastmilk only)	40 at 4wks	N/A	N/A	Maternal confidence level is related to their perception of insufficient breast milk flow.
				2. Partial breastfeeding (i.e. breastmilk and formula)				
				3. Bottle-feeding (i.e.no breastmilk at all)				
**Sucking difficulty**	Yokoyama et al. (2006) [21]	Cross-sectional	15,262	1. Breastfeeding only	N/A	N/A	N/A	Infants with poor sucking ability are 1.56 times more like to be given infant formula (95% CI = 1.12,2.18)
				2. Mixed-feeding (mainly breast with some bottle or mainly bottle with some breast)				
				3. Bottle feeding with formula milk only				
**Support from health professionals**	Sasaki et al. (2009) [23]	Longitudinal	908	1. Only breastfeeding	N/A	N/A	0.83 (0.61,1.12) (Midwives)	Without support from midwives = 1 (reference) (p = 0.83)
				2. Breastmilk and infant formula				
				3. Only infant formula				
	Kaneko et al. (2006) [20]	Cross-sectional	45,569	1. Breastfed	29.7 at 6m	1.63 (1.50,1.78)	1.76 (1.60,1.94)	Advice on child care from birth attendant/nurse
				2. Only colostrum				
				3. Not breastfeed				
**Support from husbands/partners**	Kaneko et al. (2006) [20]	Cross-sectional	45,569	1. Breastfed	21.8 at 6m	1.28 (1.21,1.37)	1.07 (1.00,1.14)	Support = advice on child care from husbands. No advice =1 (reference)
				2. Only colostrum				
				3. Not breastfeed				
	Ninomiya et al. (1997) [30]	Cross-sectional	264	1. Breastfeeding but exclusion of mix breastfeeding	37.5 at 3m	N/A	N/A	Father’s attendance to an antenatal class is correlated to full breastfeeding duration (p < 0.01).
	Ninomiya et al. (1995) [31]	Cross-sectional	719	1. Breastfeeding but exclusion of mix breastfeeding	N/A	N/A	N/A	Father’s involvements in childcare are associated with full breastfeeding duration for three months postpartum (p < 0.05).
**Family smoking environment**	Kaneko et al. (2006) [20]	Cross-sectional	45,569	1. Breastfed	22.6	0.90 (0.86,0.95)	0.92 (0.88,0.97)	Non-smoking fathers and mothers = 1 (reference)
				2. Only colostrum				
				3. Not breastfeed				
**Breastfeeding during night**	Sasaki et al. (2009) [23]	Longitudinal	908	1. Only breastfeeding	N/A	N/A	2.62(1.85,3.73)	Not breastfeeding during night =1 (reference) (p < 0.01)
				2. Breastmilk and infant formula				
				3. Only infant formula				

Note: N/A = Not Applicable, wks = weeks postpartum, m = months postpartum.

### Maternal attributes

Demographic factors that have been studied as risk factors for breastfeeding initiation and duration in Japan have included maternal age, socioeconomic status, maternal education, employment status, delivery method, parity, and smoking habits of mothers and other family members.

While higher maternal age has been associated with a longer duration of breastfeeding in most developed countries [32,33], there is no clear association in Japan. A population based study of 15,262 infants confirmed that the mean age of mothers who chose formula feeding was significantly younger than those who chose ‘full’ and ‘Any Breastfeeding’ (p < 0.001) [21]. This study was cross-sectional and analysed a database of infants aged three to six months attending for medical examinations, which has a very high response rate in Japan. In contrast, a study using data from the National Survey of 46,569 infants showed that mothers in their 30s and 40s were less likely to continue Exclusive Breastfeeding at six months postpartum than mothers in their 20s (aOR = 0.89, 95% CI = 0.84-0.94 for their 30s; aOR = 0.56, 95% CI = 0.48-0.65 for their 40s, respectively) [20]. The National Survey used a cross-sectional study design and found different results to the study by Yokoyama et al. [21]. Moreover, Sasaki et al. found no correlation between maternal age and Exclusive Breastfeeding at four months postpartum in a longitudinal study (p = 0.93, n = 908) [23]. Otsuka et al. [22] also conducted a longitudinal study (n = 262) and the results were different in that older mothers (more than 38 years old) tended to use infant formula at four weeks postpartum (p < 0.05).

Few studies have investigated associations between the duration of breastfeeding and the mothers’ socioeconomic and educational status in Japan, which may be related to privacy issues encountered in undertaking this research [34]. A study by Kaneko et al. found that mothers with a lower annual income (less than 3.9 million yen) were less likely to be exclusively breastfeeding at six months (aOR = 0.93, 95% CI = 0.88-0.99) [20]. A small study of 262 mothers found that educational level was not related to the use of infant formula at one month postpartum (p = 0.47) [22], but large studies would be needed to confirm this result.

Returning to work is a common reason given by mothers to cease breastfeeding [35]. If the mother is the main income earner, this factor is more important [36]. Kaneko et al. showed that Japanese mothers who had full-time jobs and who took childcare leave for more than six months were more likely to continue Exclusive Breastfeeding to six months than unemployed mothers (aOR = 1.14, 95% CI = 1.05-1.23) [20]. In this study, the mothers who had childcare leave for less than six months or who had not received any childcare leave had less favourable breastfeeding outcomes [20].

Mothers who smoke are less likely to initiate and to continue breastfeeding than those who are non-smokers [37]. Haku and Onishi stated that smoking mothers tended to rely more on formula feeding [38]. Similarly, in the study by Kaneko et al., fewer mothers who smoked at home were still breastfeeding at six months, compared to non-smoking parents (aOR = 0.44, 95% CI = 0.34-0.57) [20]. The risk of ceasing breastfeeding by mothers was decreased when only fathers were smokers compared to non-smoking parents (aOR = 0.92, 95% CI = 0.88-0.97) [20]. Kaneita et al. found that the mothers who did not breastfeed their infants were more likely to smoke inside their house, compared with those who were breastfeeding their infants [39]. While this study had a large sample size (n = 44,562) and used clear definitions of smoking, breastfeeding duration was not included as a variable and the definition of breastfeeding was unclear.

The majority of women in Japan choose vaginal delivery in contrast to some other Asian countries, including China and Korea, where the number of caesarean sections has increased rapidly in the past decade [40,41]. While Nakao et al. [25] and Sasaki et al. [23] analysed delivery methods as a factor that influences breastfeeding duration, the results were not significant. Associations between parity and breastfeeding duration are not consistent. In the study by Kaneko et al., multiparous women were more likely to continue Exclusive Breastfeeding for six months postpartum compared to those who were primiparous (aOR = 1.72, 95% CI = 1.63-1.81 for second delivery; aOR = 2.06, 95% CI = 1.91-2.22 for third delivery) [20]. However, other studies have not confirmed this association [22,23]. Parity was found to be associated with breastfeeding duration only in the cross-sectional study, with a larger sample size, but not in the cohort studies with smaller sample sizes.

A number of studies identified that mothers who ‘intend to breastfeed’ their infants at an early stage of pregnancy tended to have a longer duration of breastfeeding. A study in Okinawa found that mothers who intended to breastfeed their infants during pregnancy were more likely to continue breastfeeding at three months, compared with mothers undecided about feeding methods (χ^2^ = 28.3837, p < 0.01) [26]. In a further study from Tokyo and Kyoto (n = 262), mothers who intended to ‘breastfeed’, compared to those who intended to ‘exclusively breastfeed’ were more likely to have introduced infant formula at four weeks postpartum (OR = 8.6, 95% 2.5-29.5) [22]. However a recent survey of 2,722 postpartum mothers showed that only 42% were fully breastfeeding at four weeks postpartum, although during pregnancy more than 95% had intended to breastfeed their children [42]. Mothers with positive attitudes, sentiment and confidence were also more likely to continue breastfeeding [43].

Maternal confidence in their ability to breastfeed their infants, as measured by Breastfeeding Self-Efficacy Scale-Short Form, were associated with infant feeding outcome at four weeks. Otsuka et al. showed that mothers with a lower score (=< 44) of breastfeeding self-efficacy were more likely to use infant formula within four weeks (aOR = 3.5, 95% CI = 1.8-6.6) [22]. In a study using a quasi-experimental method of 117 primiparous mothers, maternal confidence levels towards breastfeeding were improved by breastfeeding education, particularly focusing on self-care programs about their breasts during breastfeeding [29]. Mothers who received this education program before hospital discharge had a higher proportion of full breastfeeding at four weeks than the control group of mothers (90% and 65%, respectively, p = 0.02) [29]. Maternal confidence levels are also found to be associated with lower levels of perceptions of breastmilk insufficiency. Otsuka et al. reported that ‘maternal perception of insufficient breastmilk’ at four weeks postpartum was significantly associated with ‘breastfeeding self-efficacy’ level at immediate postpartum periods after birth (r = 0.45, p < 0.001) [22].

Breastmilk insufficiency is a common reason given by mothers in most cultures for terminating breastfeeding and/or introducing supplementary feeding their infants [44]. A survey of 241 Japanese mothers found that at one week more than 41%, and at 4 weeks 70%, perceived that they had breastmilk insufficiency and had introduced infant formula [28]. While the participants in both studies voluntarily completed self-reported questionnaires, sample sizes in these studies were again small.

A cross-sectional study of 182 Japanese mothers investigated associations between levels of maternal attachment and infant feeding methods at three months of age. Sasano and Sumitani showed that mothers who were breastfeeding exclusively had a higher score for ‘pleasurable interaction’, a component of the maternal attachment scale, compared to those who were wholly or partly using infant formula feeding [27]. This study concluded that it is important for mothers to gain support and assistance during breastfeeding in order to increase and improve their maternal-infant bonding [27]. However this study is limited by the lack of multivariate analysis and the small sample size.

### Infants’ attributes

Factors relating to the infant are also important in breastfeeding, including low birth weight and multiple births. Low birth weight infants (less than 2500g) were less likely to be breastfed at six months (aOR = 0.67, 95% CI = 0.60-0.76) [20]. Similarly, Yokoyama et al. [21] found that mothers with multiple births were 2.44 times more likely to choose bottle feeding than those who had a singleton birth. However, the full breastfeeding rate of twins has not changed over 30 years with a study of 4,023 twins born between 1968 and 2003 showing that the rate of full breastfeeding at one month postpartum was steady at 16.8% between 1975 and 1984 and 16.7% between 1995 and 2003 [24].

### Socio-environmental attributes

Professional support in obstetric facilities is essential for mothers to improve breastfeeding rates. Kaneko et al. showed that mothers who received advice about childcare from professionals, including birth attendant/nurse, increased the duration of Exclusive Breastfeeding (aOR = 1.76, 95% CI = 1.60-1.94) [20]. Similarly, support from partners/fathers of infants was found to be important. In an early study investigating a relationship between fathers’ attendance to an antenatal class before delivery and breastfeeding duration (n = 264), Ninomiya et al. showed that for women (mothers) who continued ‘full breastfeeding’ to three months postpartum, it was more likely that their husbands would also have attended the classes, compared with those who ceased breastfeeding (63.0% and 37.0% respectively, p < 0.01) [30]. The same authors also found in another study (n = 719) that mothers who were satisfied with the involvement of fathers in childcare tended to continue full breastfeeding at three months (p < 0.05) [31]. These studies showed statistical significances using univariate analysis. Kaneko et al., using multivariate analysis, showed that mothers who discussed childcare with their partners were more likely to continue Exclusive Breastfeeding for six months (aOR = 1.07, 95% CI = 1.00-1.14) [20]. On the other hand, there was a negative association of mothers’ mothers (grandparents of infants) towards breastfeeding duration. A study reported that ‘not living with their own mothers and fathers (grandparents of infants)’ was positively associated with breastfeeding status at six months after birth, compared with those who lived with their parents (aOR = 1.14, 95% CI = 1.07-1.21) [20].

WHO and UNICEF encourage mothers to have skin to skin contact for at least 60 minutes immediately after birth and then to support early breastfeeding by ‘rooming-in’, as documented in ‘The ten steps to successful breastfeeding’ [45]. A study of 319 mothers who initiated breastfeeding within 120 minutes after birth, compared to those initiating later, found higher rates of full breastfeeding at four months postpartum (aOR = 2.5, 95% CI = 1.21-4.95) [25]. A government survey reported that 32.4% of 2,722 mothers initiated breastfeeding within 30 minutes after delivery and 17.3% of them commenced rooming-in immediately post birth [6].

## Discussion

Unlike studies from other countries [16,17], our review found that several factors, including maternal age, family income and maternal educational levels, were not associated with breastfeeding duration in Japan. Smoking status, low birth weight of infants and maternal perceptions of insufficient breastmilk supply were associated with shorter breastfeeding duration. Also, living with grandparents of infants in Japan may be an impediment to breastfeeding duration. These negative factors are keys to understanding potential targets for health promotion programs that are relevant to breastfeeding culture in Japan. One positive association that appears to differ from other countries was the association found in several studies between returning to work (child care facility provided) and continued breastfeeding [20]. This is an interesting finding that may relate to level of education and socio-economic status, but it requires further studies in different parts of the country to confirm the finding and explore the reasons for it.

Although the project, ‘Healthy and Happy Family 21 (Sukoyaka Oyako 21) was undertaken by the Japanese government, a survey of 757 community centres reported that only 10% had implemented breastfeeding programs to improve breastfeeding rates at the local level [46]. Countries that have improved breastfeeding rates have usually had a national promotion program and enlist society as a whole to support breastfeeding.

While the Japanese health system puts great emphasis on healthy development and prevention, the national breastfeeding rate at one month postpartum has remained relatively unchanged between 1980 and 2005 and appears to have slightly declined in some surveys. In Australia, a country with similar health status and level of economic development to Japan, the rate of Exclusive Breastfeeding at one month postpartum was 55.8% [47], higher than Japan at 42.4%. Different definitions of breastfeeding practices were also used in Australia and Japan. Australia uses the WHO definitions, while the latest Japanese survey (The National Survey of Infants Growth in 2010) used a definition of Exclusive Breastfeeding that was having breastmilk as the main source of nutrition and included having some infant formula when infants “go out” [13]. Consequently, much breastfeeding in Japan that is reported as Exclusive Breastfeeding is actually Predominant (full) or Any Breastfeeding and not Exclusive Breastfeeding according to the WHO definition. However, even though a less stringent definition has been used, there is still room for considerable improvement in Exclusive Breastfeeding rates to six months. This would be in accord with WHO policy and would optimise the health of Japanese infants.

In addition, some practices, including prelacteal feeding and supplemental feeding, which are not recommended by WHO, are still common in Japan. A cross-sectional study of 1,612 postpartum mothers in Himeji city reported that the mean age of starting infant formula was 2.8 weeks, which meant that the majority were classified as Any Breastfeeding after their discharge from hospital. More than 20% introduced infant formula within seven days after birth and this increased to almost 80% at two weeks postpartum [48]. In a study of 41 obstetric facilities in Okinawa prefecture (approximately 47% of the total number of the obstetric facilities), Nakamura showed that only 8 facilities (19.5%) routinely gave nothing to infants after birth except breastmilk from their own mother [26]. Nineteen facilities (46.3%) gave infant formula or glucose to infants while in hospital and five facilities (12.1%) gave these immediately after birth as their first feed. However, some authors have argued that giving glucose water as the first feed for infants prevents neonatal hypoglycaemia that may affect the neurological problems later in life [49,50]. One randomized controlled trial (RCT) found that prelacteal or early supplemental feeding was associated with shorter duration of breastfeeding, although there are no reported studies that investigated this relationship in Japan [51]. Widespread use of infant formula is observed in Japan, which is consistent with the low proportion of BFHI accredited hospitals and obstetric clinics. By 2011, it was only 2% (61 out of over 3000 hospitals and obstetrics clinics) compared to Australia, where approximately 22% (74 hospitals out of 335 obstetric/maternity hospitals) were BFHI certified in 2010 [52].

In Japan, infant formula is often introduced when infants are considered to have insufficient weight gain. This often occurs when mothers perceive that they have insufficient breastmilk production [53]. These mothers may feel guilty at not being able to breastfeed and encouraging these mothers to exclusively breastfeed their infants may subject mothers to added mental pressure and stress [50]. Health professionals also sometimes suggest to these mothers to add infant formula to relieve them from these pressures and stresses [54]. Some mothers misinterpret infants’ behaviours as a sign of insufficient breastmilk [55]. Health professionals sometimes suggest adding infant formula based on their assessment of the adequacy of breastmilk [56]. The high rate of use of infant formula for supplementary feeding is reflected in the unchanged rate of Exclusive Breastfeeding since 1980. The routine use of supplemental and prelacteal infant formula feeds is not consistent with best infant feeding practice [57]. However, Any Breastfeeding is still better for the infant than receiving only infant formula and even if mothers use some supplemental infant formula, they should continue breastfeeding as much as possible.

There are other traditional customs related to breastfeeding practices that may hinder mothers from continuation of breastfeeding in Japan. For instance, many hospitals and clinics routinely measure infants and undertake other procedures immediately after birth, which separates them from their mothers. Several studies have argued that these neonatal procedures are barriers that unnecessarily delay early initiation of breastfeeding and would in turn affect the duration of breastfeeding [25,58]. Many hospitals and obstetric clinics also accept donations of infant formula and equipment from infant formula companies and samples are sometimes distributed to new mothers while they are in hospital. Howard et al. reported that mothers who received goods from formula companies were more likely to cease breastfeeding within two weeks after birth than those who did not receive gifts [59]. A study of 151 midwives who attended an academic conference also showed that more than 45% considered that the practice of teaching new mothers to prepare infant formula not to be an obstacle to breastfeeding and 32% stated that providing free gifts of infant formula would not impede breastfeeding practices [60].

Over the past three decades, Any Breastfeeding rates in Japan have improved. The New Guidelines on Infant Feeding developed by the Japanese government in 2007 may be assisting this improvement. The new guidelines were distributed to all community health centres, encouraging all health professionals to promote breastfeeding to mothers. Health professionals can have either a positive or negative influence on the mothers’ motivation to continue breastfeeding depending on their own knowledge and attitudes and what they are prepared to promote. A study evaluating breastfeeding textbooks for Japanese midwives found that the only 40% provided accurate and consistent content on infant feeding consistent with best practice [61]. Nurses’ and midwives’ knowledge of breastfeeding would be expected to be mainly acquired from these textbooks. Mizuno et al. also reported that paediatricians (n = 90) were more aware of the importance of breastmilk than obstetricians (n = 62) (66% vs. 13%) and knowledge about the WHO marketing code was at a similar level (51% vs. 18%) [62]. Health professionals need to receive adequate education on breastfeeding knowledge and its benefits. This will enable them to deal with common concerns of mothers and will also protect mothers from confusion resulting from inconsistent information and advice in breastfeeding.

Other factors that affect breastfeeding duration including encouragement from grandmother(s) (especially, the maternal mother), husbands/partners, and other family members and society are important for continuing breastfeeding [63,64]. However, there have been few studies, particularly of fathers’ support, in relation to breastfeeding duration in Japan, although the support of fathers is acknowledged to be important for mothers to continue breastfeeding in other countries. A study of Japanese husbands that compared them with their Canadian counterparts showed they were significantly less supportive during the postpartum period in terms of giving time for their family [65]. Without such support, some Japanese women felt more isolated, and some did not even expect their husbands to participate in childcare or home duties [65]. In Japanese culture, women have a dominant responsibility for care of children and may explain the apparent lack of interest of Japanese husbands/partners in supporting breastfeeding. The 2008 National Survey on Domestic Care reported that preparing or feeding meals including infant formula to children under one year old was third on the list of supporting tasks undertaken by fathers [66]. Yokoyama and Ooki [67] found that women who had minimal support from their spouses/partners were significantly more likely to choose formula feeding (OR = 1.85, 95% CI = 1.38-2.48), although this study focused on mothers who had twin and multiple births. More research is needed on the extent of the role of fathers in supporting breastfeeding in Japan and how this could be extended. Our findings also show that support from grandmothers of infants may contribute in negative ways in breastfeeding. In other countries, the ‘mother of the mother’ has a positive influence on breastfeeding initiation and its continuation [68]. In Japanese culture, mothers often return to their hometown for their delivery and ask for help from their mothers during antepartum and postpartum periods [69]. When mothers have knowledge about the importance of Exclusive Breastfeeding, their daughters tended to have a longer duration of breastfeeding [68].

There have been a limited number of the studies on breastfeeding factors in Japan, and there are some inconsistent results between studies. Some areas of breastfeeding practices related to breastfeeding duration need to be further studied. For example, breast problems, including nipple trauma and sore nipples, are often given as reasons for discontinuation of breastfeeding by mothers, but there are few reported studies in Japan. There is a need for further cohort studies to resolve these inconsistencies. Most reported Japanese breastfeeding studies are cross-sectional in design with small sample sizes and unclear definitions of breastfeeding. One of the largest studies (n = 46,569) by Kaneko et al. used a cross-sectional design and imprecise definitions [20]. Large cohort studies are required using standard definitions of breastfeeding and including measures of breastfeeding intention, initiation and duration, together with demographic factors, physical factors, psychological factors and other potential confounders.

## Conclusion

The available studies of breastfeeding in Japan suggest that some factors related to breastfeeding duration in studies in other countries show inconsistent relationships in Japanese studies. Cultural and physical factors may be involved in breastfeeding practices, but more studies, particularly larger cohort studies, are needed to confirm any association. While Any Breastfeeding rates have improved in the last three decades, the trends in the rate of Exclusive Breastfeeding are less certain due to the use of inconsistent definitions. There is a need to standardise the way Exclusive Breastfeeding is defined in Japan. In order to further improve breastfeeding rates, there is a need for to improve the inconsistent knowledge of health professionals in Japan about infant feeding. In addition, cohort studies with larger representative samples sizes and clear definitions of breastfeeding type are needed to monitor risk factors of breastfeeding outcomes and to provide the basis for promoting breastfeeding. These will enable Japanese mothers, fathers, and health professionals to move towards the internationally recommended target of ‘Exclusive Breastfeeding for the first six months of life’.

## Abbreviations

BFHI: Baby friendly hospital initiative; WHO: World Health Organization; UNICEF: United Nations Children’s Fund; CI: Confidence interval; ORS: Oral rehydration solution; aOR: Adjusted odds ratio.

## Competing interests

The authors declare that they have no competing interests.

## Authors’ contributions

MI had primary responsibility for designing the study, searching and analysing literature and drafting the manuscript. CB was involved in revising the manuscript critically for important intellectual content. KO contributed to searching literature and revising the manuscript. MM contributed to searching and analysing literature. MJ revised the manuscript. All authors have given final approval of the version to be published. All authors read and approved the final manuscript.
